# Regional variation in prescription drug spending: Evidence from regional migrants in Sweden

**DOI:** 10.1002/hec.4552

**Published:** 2022-06-16

**Authors:** Naimi Johansson, Mikael Svensson

**Affiliations:** ^1^ Health Economics and Policy School of Public Health and Community Medicine Institute of Medicine University of Gothenburg Goteborg Sweden; ^2^ University Health Care Research Center Faculty of Medicine and Health Örebro University Örebro Sweden

**Keywords:** event‐study, pharmaceuticals, regional variation, Sweden

## Abstract

There is substantial variation in drug spending across regions in Sweden, which can be justified if caused by differences in health need, but an indication of inefficiencies if primarily caused by differences in place‐specific supply‐side factors. This paper aims to estimate the relative effect of individual demand‐side factors and place‐specific supply‐side factors as drivers of geographical variation in drug spending in Sweden. We use individual‐level register data on purchases of prescription drugs matched with demographic and socioeconomic data of a random sample of about 900,000 individuals over 2007–2016. The primary empirical approach is a two‐way fixed effect model and an event study where we identify demand‐ and supply‐side effects based on how regional and local migrants change drug spending when moving across regional and municipal borders. As an alternative approach in robustness checks, we also use a decomposition analysis. The results show that the place‐specific supply‐side effect accounts for only about 5%–10% of variation in drug spending and remaining variation is due to individual demand‐side effects. These results imply that health policies to reduce regional variation in drug spending would have limited impact if targeted at place‐specific characteristics.

## 1 INTRODUCTION

Substantial variation in health care use and costs across geographical areas have been demonstrated in several countries and is a major health policy issue (Corallo et al., 2014; Fisher et al., 2009; Skinner, 2011). Regional variation in health care spending can be justified if caused by differences in demand factors such as health needs. On the other hand, regional variation is a cause of inefficiency if driven by supply factors such as the availability of health technology and physician practices (Skinner, 2011).

It has proven difficult to empirically sort out the causes of regional variation and whether demand or supply factors are the most important remains unresolved (Cutler et al., 2019; Finkelstein et al., 2016). For example, in a region with high availability of health care providers, this could be considered a supply factor that can cause more spending. However, the high availability could result from a long‐term high demand due to large health needs.

A number of recent studies on regional variation have used regional migration data to try to separate demand and supply factors (Godøy & Huitfeldt, 2020; Molitor, 2018; Moura et al., 2019; Salm & Wübker, 2020; Song et al., 2010; Zeltzer et al., 2021). The general idea in this approach is that if place‐specific institutional features (supply) are most important, we expect regional migrants to change health care utilization behavior when moving into a new region (Finkelstein et al., 2016). On the other hand, if individual characteristics and preferences (demand) cause the regional differences, we expect migrants' health care use to remain (fairly) constant after moving.

Previous studies from various settings have estimated divergent place effects, with 9% (Salm & Wübker, 2020) to 60% (Finkelstein et al., 2016) of variation in health spending caused by supply factors. The ambiguity of results suggests that institutional settings are important for the size of the regional variation and the drivers behind the variation (Godøy & Huitfeldt, 2020; Salm & Wübker, 2020). It is not obvious on what level of aggregation regional variation should be analyzed (Zhang et al., 2012), and previous studies have used variation across provinces, hospital referral regions or postal codes (e.g., Godøy & Huitfeldt, 2020; Moura et al., 2019). Moreover, previous research has analyzed non‐drug health care spending or total health care spending. Less is known about the distribution and causes of regional variation in drug spending (Zhang et al., 2010), even as drugs alone represent about 20% of health care spending in the average OECD country (OECD, 2017).

In this paper, we run a two way fixed effects model and an event study analysis with regional migration data from Sweden to study what explains regional variation in prescription drug spending. We analyze the causes of regional variation on two levels of aggregation – across 21 regions (counties) and 290 municipalities.

We exploit prescription drug spending as the outcome of regional variation, because there is no reason to assume that the geographical pattern of variation is the same for each component of health care spending. Zhang et al. (2010) showed a weak correlation (*r* = 0.10) between drug spending and non‐drug health care spending across hospital referral regions in US Medicare, and pointed out that drugs can work as either a complement or a substitute for medical care. Moura et al. (2019) subcategorized total spending and found that place effects explained 28% of variation in prescription drug spending, and in contrast, around 21% of variation in primary care spending. It is important to understand the drivers of regional variation for each component of health spending, as the causes may differ by type of health care.

The Swedish market for prescription drugs is a good case study considering that the institutional rules may reduce the impact of supply‐side factors. In general, the single‐payer national health service system is characterized by universal coverage, low cost‐sharing, and salary‐paid physicians with minor (or no) economic incentives to over‐treat. Specific for the market of prescription drug is that physicians, hospitals, and primary health care centers have limited (if any) direct economic incentives to prescribe larger volumes or more expensive drugs than needed (in some cases even incentives to prescribe less when costs are carried by the clinic). Additionally, prescription drug prices are fixed nationally and for generic drugs, pharmacies are required to offer the cheapest generic alternative irrespective of which brand‐name drug was prescribed (Granlund, 2010).

In the rest of the paper, we show that the average regional drug spending per capita per year varies from −7% to +28% around the national mean. The documented regional variation is similar to variations in total health care spending in the Netherlands (Moura et al., 2019), but smaller than the reported regional variation in drug spending in the US (Zhang et al., 2010). The variation across municipalities is, as expected, larger, −28% to +103% around the national mean. Our results show that the place effect accounts for only about 5%–10% of the variation in drug spending in regions and municipalities, which is at the lower end of the scale compared with previous estimates. The remaining 90%–95% of the variation in drug spending is driven by an individual demand‐side effect.

Our study makes two main contributions to the literature on the causes of regional variation in health care spending. First, we provide evidence of an individual effect as the main driver of variation in drug spending. Our results emphasize the importance of institutional settings in general, but also the particular institutional rules by type of health care – indicating that place‐effects play a limited role in a national system with few incentives to over‐treat and with a generic substitution policy. Second, our results show that the level of aggregation (regions or municipalities) does not change the qualitative interpretation of our results, even though a lower level of aggregation reduce the uncertainty of our estimates.

The paper is structured as follows: in Section 2 we describe the institutional setting and data, and in Section 3 we present the empirical method. We present the results in Section 4, robustness checks in Section 5 and conclude with a discussion in Section 6.

## 2 INSTITUTIONAL SETTING AND DATA

### 2.1 Institutional setting

The Swedish national health service offers universal coverage for all residents. The system is decentralized in 21 regions with responsibility to finance and provide health care. The regions subdivide into 290 municipalities with responsibility (among other things) for long‐term care. The provision of health care is carried out by a mix of public and private providers and all providers are reimbursed at the same rate through public funds (regional and municipal income taxation).

Health care is subsidized at point of service with relatively small out‐of‐pocket prices for health services, identical across providers within the same region (private and public). The cost‐sharing scheme for prescription drugs is identical for all regions and takes the form of a deductible with multiple thresholds, where the patient annually pays a maximum of €224 (1€ = 10.5 SEK, year 2019). The patient out‐of‐pocket price for prescription drugs is the same irrespective of whether the physician is employed in public or private. The national Dental and Pharmaceutical Benefits Agency (TLV), regulates which drugs are included in the national pharmaceutical benefits scheme based on health need, disease severity, and cost‐effectiveness (Svensson et al., 2015). If a drug is approved, it is sold at private pharmacies throughout the country at the fixed price agreed by the producer and TLV. In 2009, the pharmacy market was deregulated from a single state‐owned pharmacy to allow for multiple private owners, which lead to an increase by 22% in the number of pharmacies (Anell et al., 2012; Swedish competition authority, 2010).

### 2.2 Sample

We base our analysis on a random sample of 1 million individuals of the Swedish population, followed over 10 years (2007–2016). After excluding children under 15, the sample consists of 929,711 individuals and about 8.2 million individual‐year observations. The data set contains details on all purchases of prescribed drugs matched with demographic and socioeconomic background statistics at the individual level. The data have been collected from the National Board of Health and Welfare's register of prescribed drugs and population registers of Statistics Sweden and merged using individual identification numbers.

Key variables for our analyses are total drug expenditures per year, that is, the sum of the cost for the payer (the region) and the patient's out‐of‐pocket costs; and the place of residence. We assess regional variation across the 21 Swedish regions and the 290 municipalities motivated by the organizational structure and data availability. The regional ethics review board in Gothenburg approved the merging of registers and the analysis plan (#803‐17).

### 2.3 Drug spending at the individual level

Drug spending per capita is highly right‐skewed. In the entire sample, almost 30% of observations are zeros (Table 1). The mean drug spending per capita and year is €319 and the median is €44. The highest cost per patient and year is above €1.6 million.

**TABLE 1:** Distribution of drug spending on the individual level

		Regional level		Municipal level	
	Full sample	Non‐migrant	Migrants	Non‐migrants	Migrants
Individuals	929,711	843,965	53,620	756,385	102,943
Ind.‐year obs.	8,242,510	7,426,514	507,510	6,587,919	977,359
Drug spending per capita per year (€)					
Mean	319.25	332.06	221.74	343.78	240.52
SD	2607.68	2677.78	1638.81	2686.24	1750.24
Median	44.29	48.10	24.76	52.38	27.48
Min	0	0	0	0	0
25th –75th perc	0 – 209.52	0 – 226.71	0 – 110.10	0 – 244.71	0 – 122.62
Max	1,617,142.88	1,617,142.88	263,676.38	1,617,142.88	289,899.16
Share obs. With zero costs	28.87%	28.05%	35.08%	27.38%	33.45%

*Note*: A migrant is defined as a person who moves once between regions or between municipalities in 2008–2015. Individuals who move more than once are excluded from the analysis. €1 = 10.5 SEK (2019).

We base our identification strategy on individuals who move across region borders. We define regional migrants as individuals who move between regions once during the study period and where we can follow spending both before and after the move (i.e., moves that occurred between 2008 and 2015). The sample of movers consists of 53,620 regional migrants and 507,510 migrant‐year observations (Table 1). The sample almost doubles when we include individuals that move across municipal borders; 102,943 municipal migrants and 977,359 migrant‐year observations. The migrants differ from non‐migrants; the most notable differences are that migrants are younger (mean age 36 vs. 50 years), have a higher education level, a higher proportion is unmarried, and the migrants have considerably lower drug spending (Table 2).

**TABLE 2:** Background statistics on non‐migrants and migrants

	Regional level		Municipal level	
	Non‐migrant	Migrants	Non‐migrant	Migrants	
Women (%)	50.77	50.66	50.97	49.91	
Education level (%)					
Tertiary education	27.87	37.94	27.47	35.85	
Upper secondary school	43.54	38.07	43.6	40.92	
Primary + lower second. School	27.01	22.59	27.35	21.86	
Missing	1.57	1.4	1.58	1.37	
Employed (%)					
Yes	59.46	57.49	58.75	63.72	
No	40.54	42.51	41.25	36.28	
Marital status (%)					
Married	44.01	24.62	46.13	29.18	
Unmarried	36.95	63.48	34.11	57.23	
Divorced	11.86	9.76	11.95	11.11	
Other	7.18	2.14	7.8	2.48	
Children at home (%)					
Yes	35.21	35.6	34.5	37.9	
No	64.79	64.4	65.5	62.1	
Age, years (Mean, SD)	49.50 (19.08)	35.55 (17.08)	51.12 (18.76)	37.89 (17.03)	
Yearly disp. Income (Mean, SD)					
Individual, thousand €	19.015 (48.669)	15.711 (24.632)	19.220 (50.412)	17.757 (30.810)	
Household, thousand €	35.308 (201.438)	33.124 (40.320)	35.240 (209.166)	35.682 (110.158)	

*Note*: Statistics shown as proportions and for the continuous variables, as mean and standard deviation. All figures are based on the individuals' status 2007. A migrant is defined as a person who moves between regions or municipalities once in 2008–2015.

### 2.4 Drug spending on the regional level

**FIGURE 1:**
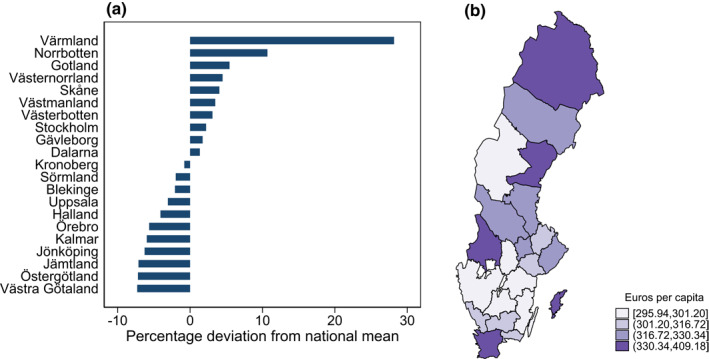
Percentage deviation and geographical pattern – regions. Panel (a) shows the percentage deviation from the national mean, where zero on the *x*‐axis represents the mean national spending of prescribed drugs per capita per year and the horizontal bars show the percentage deviation in average regional drug spending. The national (weighted) mean was €319 (€1 = 10.5 SEK 2019). Panel (b) shows the geographical pattern of mean regional spending, in euros per capita. The averages in (a) and (b) are pooled over the years 2007–2016, using the full sample of 929,711 individuals.

**FIGURE A1:**
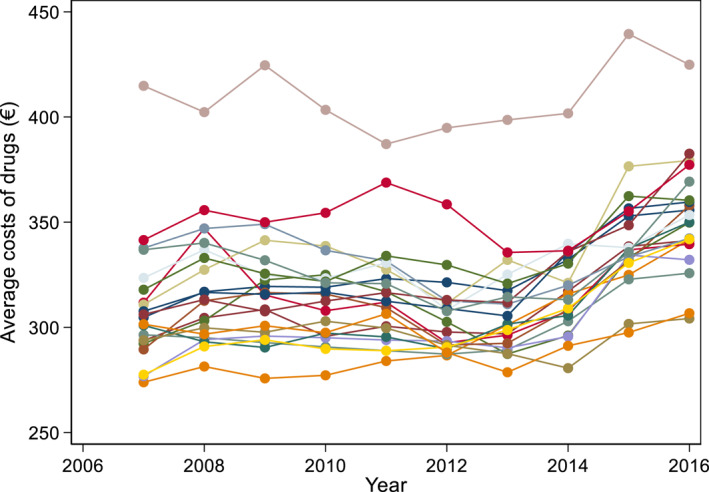
Development over time of average regional drug spending. The lines represent the regional mean spending of prescribed drugs per capita per year for the 21 regions. The (weighted) national mean over 2007–2016 was €319 (€1 = 10.5 SEK 2019). Spearman’s correlations estimated between 0.72 and 0.96 over each pair of the ten years

Across the 21 Swedish regions (NUTS 3, Eurostat European Commission, 2018), average regional drug spending per capita per year varies from −7% to +28% around the national mean (Figure 1). The size of variation is similar to that of variations in total health care spending in the Netherlands' provinces (Moura et al., 2019). Expressing the variations in terms of the ratio of highest to lowest, the ratio of 1.38 (Table 3) is smaller than the variation in drug spending in US Medicare (ratio 1.6) (Zhang et al., 2010). Figure A1 in the Appendix shows that regional averages are stable over the study period (Spearman's correlations estimated between 0.72 and 0.96 over each pair of the 10 years).

**TABLE 3:** Distribution of drug spending on the regional and municipal level (€)

	Regional level	Municipal level
Lowest	295.94	230.85
25th percentile	301.20	292.13
Median	316.72	317.80
75th percentile	330.34	344.10
Highest	409.18	647.72
Mean (unweighted)	321.25	325.29
Standard deviation	25.69	52.58
Ratio highest/lowest	1.38	2.81
Ratio75th/25th	1.10	1.18
Coefficient of variation	0.08	0.16

*Note*: Mean spending of prescribed drugs per capita per year (€) of 21 regions and 290 municipalities, respectively. The averages are pooled over the years 2007–2016. The coefficient of variation is calculated as the ratio of the standard deviation to the mean. €1 = 10.5 SEK (2019).

**FIGURE 2:**
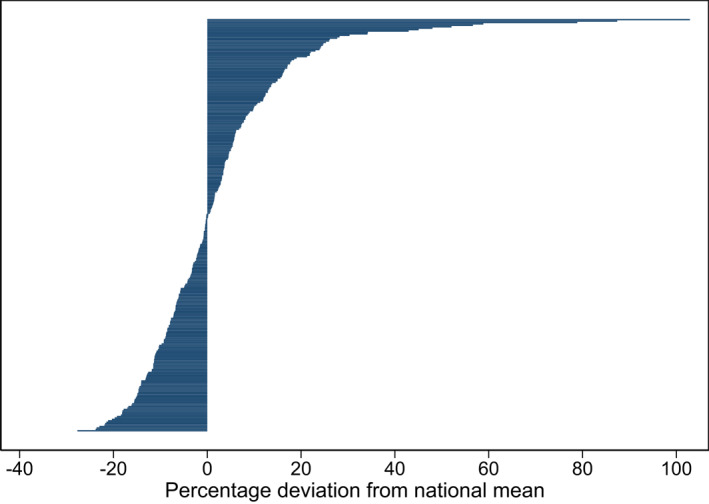
Percentage deviation from national mean – municipalities. Zero on the *x*‐axis represents the mean national spending of prescribed drugs per capita per year and the horizontal bars show the percentage deviation in average municipal drug spending. The averages are pooled over the years 2007–2016, using the full sample of 929,711 individuals. The full sample's national (weighted) mean was €319 (€1 = 10.5 SEK 2019)

The variation across municipalities is, as expected, larger, varying between −28% and +103% around the national mean (Figure 2). Regional variation in drug spending is only weakly correlated to gross regional product (Spearman's correlation coefficient of 0.20 over years 2007–2016, results available on request).

## 3 EMPIRICAL APPROACH

**FIGURE A2:**
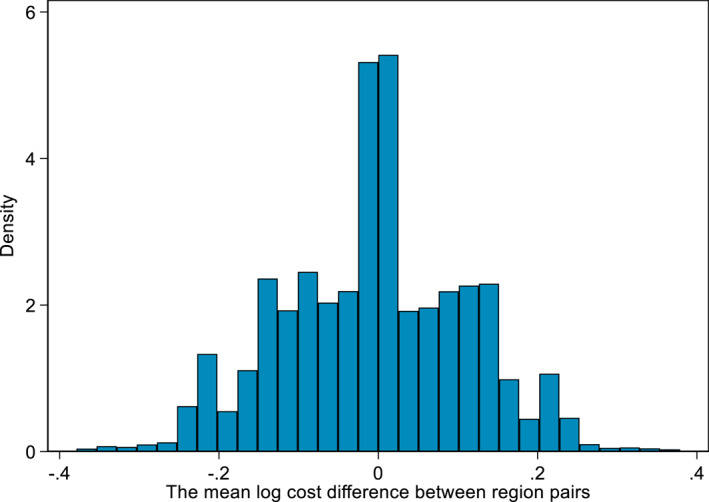
Distribution of *D*_*i*_ for migrants across regions. The distribution of the migrants’ D‐values, is fairly symmetrical around 0, indicating that moving from a high‐consumption region to a low‐consumption region is as common as vice versa. This supports the assumption that moving to a different region is exogenous with respect to the consumption of prescribed drugs

Our main approach to differentiate between demand‐ and supply factors is a two‐way fixed effect model with individual and year fixed effects, and an event study specification (Finkelstein et al., 2016). We use an alternative decomposition approach as a robustness check, described further in Section 5. The idea behind the main analysis is to see how drug spending changes when an individual move to a different region (municipality) with different supply‐side characteristics and spending levels. The analysis only includes individuals in the sample who moved between regions (municipalities) during the study period. The two‐way fixed effect equation that we estimate is:

(1) $$y_{it}=D_{i}I_{t>r}\theta +X_{it}\beta +I_{R}+\tau_{t}+\alpha_{i}+\varepsilon_{it}$$

where $y_{it}$ is the log drug spending of individual *i* in year *t*. The main independent variable of interest is the difference in mean log drug spending between the region of origin and the region of destination:

(2) $$D_{i}={\bar{y}}_{j_{dest}\;\left(i\right)}–{\bar{y}}_{j_{orig}\;\left(i\right)}$$

for individual *i* who moves from region $j_{orig}$ to region $j_{dest}$. The regional mean log spending is the pooled mean over the 10 years $\left({\bar{y}}_{j}=\left[\sum_{t=1}^{10}{\bar{y}}_{jt}\right]/T\right)$ for region *j*. Using the mean of log spending, $D_{i}$ is approximately the percentage difference in spending between the two regions $j_{orig}$ and $j_{dest}$. With 21 regions, $D_{i}$ can take up to 21*×* 20 = 420 distinct values (with 290 municipalities $D_{i}$ can take up to 83,810 distinct values). Figure A2, A3 in the Appendix show that the distributions of the migrants' $D_{i}$‐values for regions and municipalities are approximately symmetric, implying that moves from high‐ to low‐consumption regions (municipalities) are as common as moving from low to high‐consumption regions (municipalities). This supports the assumption that moving to a different area is exogenous with respect to the consumption of prescription drugs.

The binary indicator $I_{t>r}$ takes the value 1 in years after the move (t>r), and 0 otherwise. The main parameter of interest is θ, which will reflect the (percentage) change in individual spending in the years after the move, given the difference in average log spending between origin and destination region $\left(D_{i}\right)$. $X_{it}$ is a vector of time‐varying individual characteristics and β is a vector of parameters to be estimated. Included individual‐level variables are: binary indicators for gender‐specific 10‐year age groups (women 30–39, men 40–49, etc.), individual disposable income, and family situation defined by marital status and number (and age) of children in the household. $I_{R}$ is a vector of binary indicators for years since the move, where *r* is the number of years after the move, accounting for effects of migration that are unrelated to D ($I_{1}$ takes the value 1 for year one after the move and 0 otherwise, $I_{2}$ takes the value 1 for year two after the move and 0 otherwise, etc.). Additionally, $\tau_{t}$ is year fixed effects; $\alpha_{i}$ is individual fixed effects, and $\varepsilon_{it}$ is an error term that represents unobserved individual characteristics.

θ is the share of variation attributed to a place effect (Salm & Wübker, 2020). If θ=1, the difference in region average spending completely predicts individual spending changes at the time of a move, adjusted for changes in included individual‐level covariates, and variations are driven by regional “supply” characteristics. If θ=0, the difference in region average spending does not affect individual spending, assuming that individual “demand” characteristics cause the variations. We expect θ to have a value between 0 and 1, such that the place effect explains regional variation in part (𝜃), and the individual effect explains the remaining part (1−𝜃).

To draw causal conclusions of θ, the following exogeneity assumption must hold:

(3) $$E\left(\varepsilon_{it}|D_{i}I_{t>r},X_{it},\tau_{t},I_{R}\right)=0$$

The assumption requires that the explanatory variables, such as $D_{i}$, are unrelated to unobserved individual‐level time‐varying characteristics ($\varepsilon_{it}$). The expression makes no assumption about $a_{i}$, which implies that a potential association between $D_{i}$ and time‐invariant unobserved factors, such as stable patient preferences, does not violate the causal interpretation of θ (Salm & Wübker, 2020). Something that may violate the exogeneity assumption is if unobserved time‐varying characteristics are systematically correlated to $D_{i}$ or to other included covariates. That could potentially arise if individuals experiencing a negative health shock tend to move to regions with higher drug spending, if the effect of $D_{i}$ is nonlinear or asymmetric (e.g., different impact on drug spending depending on if moving to a high‐ or low‐consumption region) or if θ varies over time (Salm & Wübker, 2020).

To assess potential spending trends in years before and after the move, we estimate year‐specific θ’s in the following event study regression:

(4) $$y_{it}=D_{i}I_{r}\theta_{r}+X_{it}\beta +I_{R}+\tau_{t}+\alpha_{i}+\varepsilon_{it}$$

where $D_{i}$ is interacted with binary indicators $I_{r}$ for each year before and after the move, allowing the effect of $D_{i}$ to differ each year. We set the coefficient for the year before the move (r=−1) to zero. Concerning our relatively small sample for regional migrants, we restrict the binary indicators $I_{r}$ to years −5≥r≤5 around the move, but include all available years around the move (−8≥r≤8) for the analysis of municipal migrants. The estimated model in Equation (4) tests whether there are systematic changes in log drug spending pre‐ and post‐move; while the main Equation (1) assumes that the pre‐trend is flat.

We run the two‐way fixed effects regression with ln(spending+1) as the main outcome, with and without independent variables of individual characteristics. Due to many zeros in drug spending, we assess the robustness of our results and run the model with various forms of the dependent variable, namely ln(spending+2), ln(spending+10) and ln(spending), as well as the inverse hyperbolic sine transformation (arcsinh) $\ln\left(spending+\sqrt{spending^{2}+1}\right)$ (Bellemare & Wichman, 2020). We run this set of analyses first based on variation and migration across regions and second based on variation and migration across municipalities. To further assess the importance of skewness in drug spending, as a small share of individuals have very high costs of drugs, we create a 95‐percentile trimmed sample where we exclude the top 5% of observations. A thorough description of the trimmed sample can be found in the Supporting Information.

## 4 RESULTS

The place effect $\hat{\theta }$ is in the analysis of regions estimated to 0.05 with a confidence interval of −0.11 to 0.21 (Model 2 in Table 4). Results are similar irrespective of excluding or including the individual‐level independent variables in the model specification (Model 1 vs. Model 2). In alternative models, $\hat{\theta }$ is estimated between −0.03 and 0.06. Running the analyses of variation across municipalities, we estimate a place effect of 0.10 (CI 0.04; 0.16) in the preferred specification (Model 2 in Table 5), and $\hat{\theta }$’s between 0.03 and 0.11 in alternative specifications.

**TABLE 4:** Results of the two‐way fixed effect regressions – variation across regions

	Model 1	Model 2	Model 3	Model 4	Model 5	Model 6
Dependent variable	ln(spend+1)	ln(spend+1)	ln(spend+2)	ln(spend+10)	ln(spend)	arcsinh
$\hat{\theta }$	0.049	0.050	0.040	0.016	−0.027	0.060
st.err.	0.083	0.083	0.081	0.075	0.082	0.084
95% C.I.						
lower limit	−0.114	−0.112	−0.118	−0.131	−0.187	−0.106
upper limit	0.211	0.212	0.198	0.164	0.133	0.225
No of ind‐year obs.	507,510	507,510	507,510	507,510	329,463	507,510
No of ind.	53,620	53,620	53,620	53,620	51,298	53,620
Independent vars.^a^	No	Yes	Yes	Yes	Yes	Yes
Years since move	Yes	Yes	Yes	Yes	Yes	Yes
Year FE	Yes	Yes	Yes	Yes	Yes	Yes

*Note*: All fixed effects regressions are run with the sample of 53,620 migrants over the years 2007–2016. In Model 1, the regression is run without independent variables of individual characteristics. In Model 5, only positive costs are included. Full regression results are available on request.

^a^ Independent variables in Model 2–6 include indicators for age‐gender group, individual income, marital status, and the number of children in the household.

**TABLE 5:** Results of the two‐way fixed effect regressions – variation across municipalities

	Model 1	Model 2	Model 3	Model 4	Model 5	Model 6
Dependent variable	ln(spend+1)	ln(spend+1)	ln(spend+2)	ln(spend+10)	ln(spend)	arcsinh
$\hat{\theta }$	0.098	0.102	0.096	0.082	0.032	0.106
st.err.	0.030	0.030	0.029	0.028	0.038	0.031
95% C.I.						
lower limit	0.040	0.043	0.039	0.028	−0.041	0.046
upper limit	0.156	0.160	0.154	0.136	0.106	0.166
No of ind‐year obs.	977,359	977,359	977,359	977,359	650,465	977,359
No of ind.	102,943	102,943	102,943	102,943	98,860	102,943
Independent vars.^a^	No	Yes	Yes	Yes	Yes	Yes
Years since move	Yes	Yes	Yes	Yes	Yes	Yes
Year FE	Yes	Yes	Yes	Yes	Yes	Yes

*Note*: All fixed effects regressions are run with the sample of 102,943 migrants across municipalities over the years 2007–2016. In Model 1, the regression is run without independent variables of individual characteristics. In Model 5, only positive costs are included. Full regression results are available on request.

^a^ Independent variables in Model 2–6 include indicators for age‐gender group, individual income, marital status, and the number of children in the household.

We emphasize that a place effect of zero would indicate that the differences in regional (municipal) average spending do not affect individual spending after moving to a new region (municipality). We interpret 1− $\hat{\theta }$ as the individual effect hence, we find an individual effect around 0.90–0.95 on region and municipal level.

The results on region and municipal level are similar, with narrower confidence intervals in the analysis on municipal level (due to the larger sample of migrants). With an upper limit of the confidence interval of 0.21 in the preferred specification of the region analysis (Model 2 in Table 4) and 0.16 in the municipal level analysis (Model 2 in Table 5), the place‐specific supply‐side effect is likely substantially less important than the individual demand‐side effect for variation in drug spending.

Running the analysis on the sub‐period 2010–2016, that is, after the deregulation of the pharmacy monopoly, the place effect of is similar to the main results (Table A1, A2 in the Appendix). The variation across regions is estimated between −0.07 and −0.04, and for variation across municipalities the place effect is estimated between 0.01 and 0.10. Running the analysis with a trimmed sample and variation across regions yield results similar to the main analyses, with estimates of $\hat{\theta }$ ranging from −0.06 to 0.09 with wide confidence intervals overlapping zero (see Table S5 in Supporting information).

**FIGURE 3:**
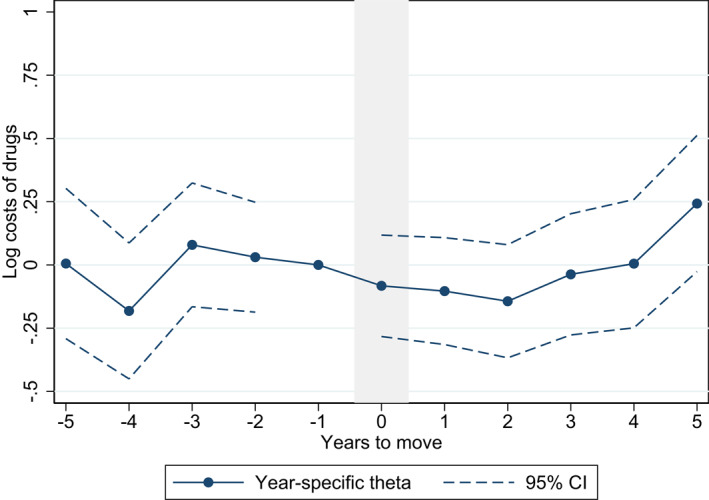
Event study of pre‐ and post‐move trends – variation across regions. Estimated based on Model 2 with ln(spending+1) as the dependent variable and including independent variables of individual characteristics. The year‐specific thetas are restricted to 5 years before and 5 years after the move

**FIGURE 4:**
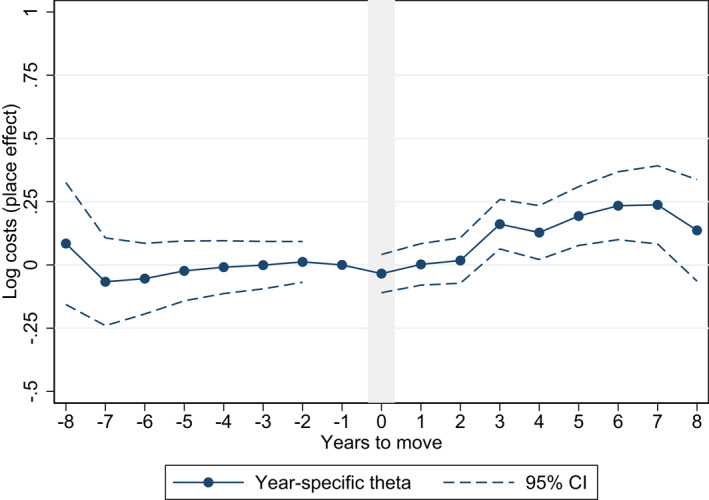
Event study of pre‐ and post‐move trends – variation across municipalities. Estimated based on Model 2 with ln(spending+1) as the dependent variable and including independent variables of individual characteristics

Estimating spending trends with year‐specific $\hat{\theta }$’s in the event study specification, evidence of a pre‐trend would suggest an over‐estimation of the place effect in the main Equation (1). However, we do not find evidence of pre‐trends in the years before the move (Figures 3 and 4). Altogether, we find limited evidence for a positive place effect after the move in the region‐level analysis, as only one of the post‐move year‐specific $\hat{\theta }$’s has a positive point estimate (five years after the move).

There is high uncertainty in the analysis of regions with relatively wide confidence intervals overlapping zero. In the event study analysis on municipal level however, we find evidence of a positive place effect that is seen not immediately but about three years after the move. Overall, the event study results confirm that the place effect is limited or small, and that the individual effect is driving variation in drug spending.

## 5 ROBUSTNESS CHECKS

### 5.1 Decomposition analysis

As a robustness check, we use a three‐way fixed effects model with individual, region, and year fixed effects in a decomposition analysis (Finkelstein et al., 2016). The decomposition analysis includes migrants and non‐migrants, but the identification relies on the migrants; without individuals moving across regions, it would be impossible to separate individual fixed effects from region fixed effects. We assume that drug spending is a product of observed individual characteristics, unobserved region and individual characteristics, and time effects. The decomposition analysis equation is specified as:

(5) $$y_{ijt}=X_{it}\beta +\rho_{j}+\tau_{t}+I_{R}+\alpha_{i}+\varepsilon_{ijt}\text{,}$$

where $y_{ijt}$ is the log drug spending of individual *i* in region *j* in year *t,* $\rho_{j}$ is region fixed effects, $\varepsilon_{ijt}$ is an error term that represents time‐varying individual characteristics, and the rest is defined as above.

The estimated region fixed effects' coefficients from this regression form the basis for the decomposition. When estimating the region fixed effects, $\rho_{j}$, one region has to serve as the reference case and the coefficients are only relevant in comparison to each other, which implies that we choose two regions or two groups of regions for comparison in the decomposition. We define regions above median drug spending as group A (high‐consumption regions) and regions below median drug spending as group B (low‐consumption regions). In an alternative decomposition, we consider the comparison between regions in the top quartile as group A and regions in the bottom quartile as group B. The difference in average drug spending between group A and B is decomposed into one part attributed to place and one part attributed to individuals, as seen in the equation:

(6) $${\bar{y}}_{A}-{\bar{y}}_{B}=\left({\bar{\rho }}_{A}-{\bar{\rho }}_{B}\right)+\left({\bar{\varphi }}_{A}-{\bar{\varphi }}_{B}\right)$$

Where ${\bar{\rho }}_{A}$ and ${\bar{\rho }}_{B}$ are the mean place effects in groups A and B respectively and ${\bar{\varphi }}_{A}$ and ${\bar{\varphi }}_{B}$ are the mean individual effects in respective groups (however, the $\bar{\varphi }:s\;$ are not estimated in the regression model). Rearranging Equation (6), the share of drug spending variation attributed to place is estimated as:

(7) $$S_{region}=\frac{{\bar{\hat{\rho }}}_{A}-{\bar{\hat{\rho }}}_{B}}{{\bar{y}}_{A}-{\bar{y}}_{B}\text{,}}$$

where ${\bar{\hat{\rho }}}_{A}$ and ${\bar{\hat{\rho }}}_{B}$ are the mean of the estimated region fixed effects' coefficients in group A and B, respectively, and ${\bar{y}}_{A}$ and ${\bar{y}}_{B}$ are the (unweighted) mean of the actual drug spending in the same groups. From this follows that the share of regional variation attributed to individuals is estimated as $S_{ind}=1-S_{region}$. Confidence intervals for the place share and the individual share are estimated by bootstrapping the sample with 250 bootstrap replicates. We use clustered bootstrap sampling on individual level, so for each individual drawn, the whole cluster of yearly observations is used. We use the 2.5th and 97.5th percentiles to form 95% confidence intervals.

### 5.2 Robustness checks results

**FIGURE A4:**
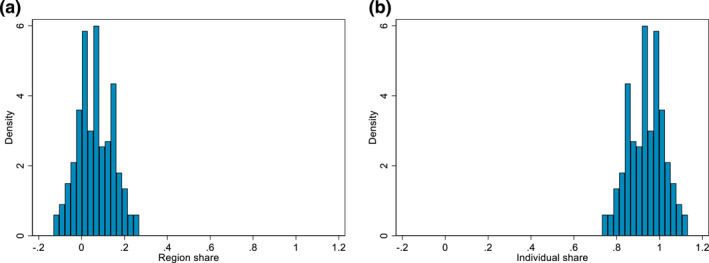
Histogram of bootstrapped place (region) and individual shares. Estimated region and individual shares from 250 bootstrapped samples, running regression based on “Model 2” and decomposed comparing regions above/below the median. (Graphs for other models/decompositions/sample available on request)

In the decomposition analysis comparing regions above and below the median, we find a share of 0.07 of regional variation attributed to place‐specific supply factors, with a bootstrapped confidence interval of −0.10 to 0.23 (Model 2 in Table 6). Figure A4 in the Appendix shows the distribution of the bootstrapped place and individual shares. Comparing the top and bottom quartile of regions, the point estimate of the place effect is 0.11 (lower panel of Table 6). We note that the results deviate slightly in the simpler model without independent variables (Model 1), estimating a place effect of 0.28 but with a wider confidence interval. The results from the decomposition are in line with our main results and show that individual‐level characteristics outweigh place‐specific characteristics as the main drivers of regional variation. The results from alternative model specifications are similar to the main results (Table A3 in the Appendix).

**TABLE 6:** Results from the decomposition analysis

	Model 1	Model 2
Dependent variable	ln(spend+1)	ln(spend+1)
Comparing regions above and below the median		
Difference in average log spending	0.153	0.153
Place share	0.050	0.072
95% CI	−0.123; 0.203	−0.098; 0.228
Individual share	0.950	0.928
95% CI	0.797; 1.123	0.772; 1.098
Comparing top and bottom quartile of regions		
Difference in average log spending	0.252	0.230
Place share	0.279	0.107
95% CI	−0.064; 0.621	−0.037; 0.252
Individual share	0.721	0.893
95% CI	0.379; 1.064	0.748; 1.037
Independent vars.	No	Yes
Year FE	Yes	Yes
Region FE	Yes	Yes
Years since move	Yes	Yes
No of ind‐year obs.	8,242,510	8,242,510
No of ind.	929,711	929,711

*Note*: The effect shares are estimated in fixed‐effects regressions. For each model, the decomposition is estimated comparing regions above/below median spending and regions above p75/below p25 of spending. Confidence intervals are estimated by bootstrapping with 250 repetitions drawn at the individual level and composed by 2.5 and 97.5 percentile of the bootstrap estimates. In Model 1, the regression is run without independent variables of individual characteristics. In Model 2, independent variables include indicators for age‐gender group, individual income, marital status, and the number of children in the household.

## 6 DISCUSSION

In this paper, we have estimated the relative effect of individuals and place on the variation in drug spending. In our main analysis, we estimate that the place effect accounts for about 5%–10% of the variation in drug spending. The results indicate that most of the variation in drug spending is caused by individual‐level demand factors, both concerning regional and municipal level variation. Robustness checks using a decomposition analysis support our results.

There is only one study, to our knowledge, that has estimated the relative effect of individuals and place in regional drug spending variation using a similar approach: Moura et al. (2019) estimated a place effect of 28% for variation in prescription drug spending, indicating a more prominent supply‐side effect compared to our results. A larger place effect was also found for regional variation in US Medicare health service spending and in Norwegian hospital spending at about 50% (Finkelstein et al., 2016; Godøy & Huitfeldt, 2020). Our point estimates are closer in magnitude to variation in outpatient services in Germany, where Salm and Wübker (2020) estimated a place effect of about 10–20%.

As noted by other authors, current evidence strongly suggests that the causes of regional variation differ depending on institutional setting, but also by type of care. The relatively small place effect found in German outpatient care, in line with our results, was interpreted as a result of high restrictions on physicians combined with many available choices for patients (Salm & Wübker, 2020). The larger place effect found in Norwegian hospital spending was argued to be reasonable given the context of the demographic, geographic, and environmental conditions in Norway with low population density and long travel times (Godøy & Huitfeldt, 2020).

The Swedish setting has several aspects similar to the German and the Norwegian, such as low cost‐sharing and regulations on physicians' treatment alternatives. But relative to Germany, patients in Sweden have fewer options for choosing provider (varying by type of care and where in the country the patient lives). In the setting of prescription drugs, one of the main features that likely affect costs of drugs, is that physicians have limited, or no, economic incentive to “over‐prescribe”. Together with fixed prices on national level and pharmacies obligation to offer the cheapest generic when available (Granlund, 2010), these regulations likely limit both the size of regional variation and the scope of place‐specific supply factors as drivers of variation.

We extend the analysis compared to previous papers by assessing geographical variation on region and municipal level, and find similar results on both levels of aggregation. Using a lower level of aggregation, implies more variation in the main independent variable in two aspects: First, expected variation is larger across municipalities than regions (the magnitude of $D_{i}$), and second, the number of potential values of $D_{i}$ is multiplied manifold. Additionally, the larger number of migrants across municipalities reduces the uncertainty of our results. Finding a relatively small place effect both on regional and municipal level indicates that neither region‐specific nor municipal‐specific supply‐side conditions affect drug spending to a major extent.

The main empirical approach used in this study, differs from the decomposition assessed in the robustness checks, even though both methods aim to estimate the share of regional variation driven by a place effect. In the decomposition analysis, using both migrants and non‐migrants, the place effect is measured by how much of average spending is captured in the estimated region fixed effects. In the two‐way fixed effect model, this is done by estimating how much average regional spending affects individual level spending at the time of a move, based on regional migrants only. All available pair combinations of a region of origin and destination region are considered in the main analysis and the estimated place effect will rely more heavily on regions with more frequent migrations. In the decomposition analysis, on the other hand, the choice of regions to compare becomes crucial as each region is given the same weight in the exercise regardless of the region's population size or the number of migrants. This raises the question of what regions are most relevant to base the analysis of regional variation on, regions with more frequent migration, or the most extreme regions in the top and bottom that account for the major part of variations, or defined by some other measures.

One of the major determinants of prescription drug use is medical need or, in other words, individual health. A limitation in our analysis is that we do not adjust drug spending for individual health, for example, with a comorbidity index. However, assuming individual health status remains fairly constant over the time period, the included individual fixed effects will account for time‐constant comorbidities.

In this study we assess regional variation in total drug spending and find limited scope of a place effect, however, the effects on total drug spending may hide heterogeneity with respect to specific drugs or ATC groups. Studies on implementation of drugs in Sweden have shown a larger variation across regions for example, about a 4‐fold variation in drugs for heart failure and for MI‐prevention (Fu et al., 2020; Johannesen et al., 2020), and it remains to be investigated whether the place‐effect has a more prominent role for certain types of drugs.

There are some potential violations of the exogeneity assumptions in the main analysis that could limit the causal interpretation of the results (Salm & Wübker, 2020). One concern would be if patients react to a negative health event by moving to a region with higher spending. However, we consider it unlikely that people because of bad health would move to regions with higher drug spending since knowledge and information about drug spending is likely incomplete. Further, if patients are aware of physician preferences and generosity regarding prescriptions, a plausible reaction would perhaps be to see a different physician in the home municipality and region than to move.

Another violation of the exogeneity assumption would be if θ varied over time, for example, if a policy reform shifted the relative effect of individual and place. In 2012, the annual cost‐sharing maximum was raised, changing the economic incentives for patients. The change was uniform across all regions. The increase in the number of pharmacies following the deregulation of the pharmacy market in 2009 also seem to have had a limited effect on our results, as seen in the point estimates from analyses of the sub‐period 2010–2016.

A third potential violation is if the effect of $D_{i}$ on drug spending is nonlinear and varies depending on moving to a high‐ or low‐spending region. The results from the decomposition analysis suggest that the relative effect of individual and of place differs depending on what regions are being compared.

**FIGURE A5:**
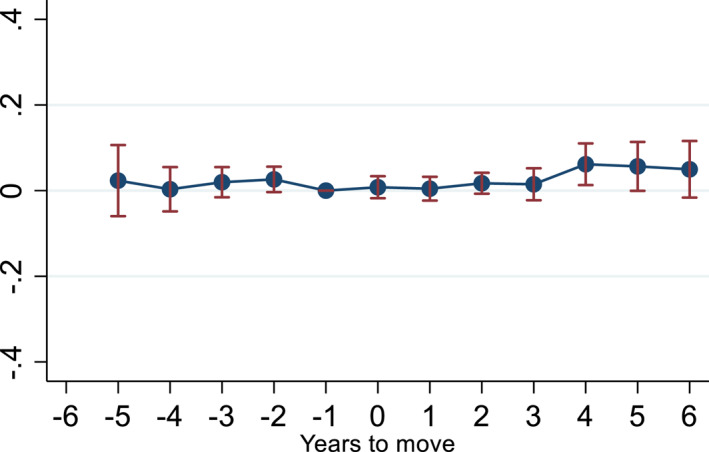
Event study regression, robust to heterogeneous treatment effects. Running our analysis of variation across regions with an alternative estimator robust to heterogeneous treatment effects (estimated by the Stata package did_multiplegt). Standard errors by 100 bootstrap repetitions

Recent discussions on the use of two‐way fixed effects models of panel data with variation in treatment timing imply that we should be cautious in terms of interpreting the results as an average place effect (Callaway & Sant’Anna, 2021; Goodman‐Bacon, 2021; Sun & Abraham, 2021). The two‐way fixed effects model estimates a weighted average of the treatment effect considering all possible pairs of treated and untreated units at different time points (Goodman‐Bacon, 2021). Researchers have suggested different ways to deal with heterogeneity in the treatment effect over time and across groups in a binary treatment setting (Callaway & Sant’Anna, 2021; Sun & Abraham, 2021) and for continuous treatments (de Chaisemartin et al., 2022). Running an additional event study analysis with an alternative estimator robust to heterogeneous treatment effects (de Chaisemartin & D'Haultfoeuille, 2020; de Chaisemartin et al., 2022) yield results similar to our main analyses and does not change the overall conclusion of our results (Figure A5 in the Appendix). Even though we cannot rule out bias in interpreting our main results as an average treatment effect, this additional analysis indicate that the likelihood for a bias of a relevant magnitude is in this case small.

In conclusion, our findings show that individual‐level demand‐side characteristics are the main drivers of regional and municipal variation in prescription drug spending in Sweden. The results imply that health policy with the aim to reduce regional variation would have limited impact if targeted at place‐specific supply‐side characteristics. Future research should study the causes of regional variation concerning the different sub‐components of health care spending or utilization, and which individual‐level demand‐side factors, such as health need or socioeconomic status, are most the important drivers of variation.

## CONFLICT OF INTEREST

None.

## Supporting information

Supporting Information S1

## Figures and Tables

**FIGURE A3:**
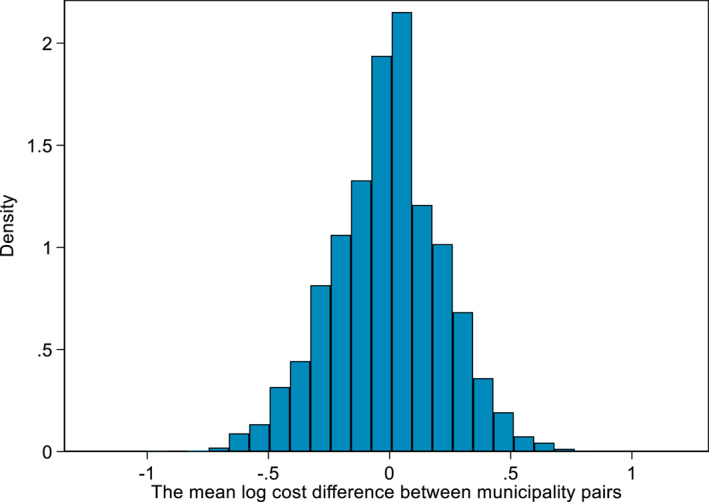
Distribution of *D*_*i*_ for migrants across municipalities. The distribution of the migrants’ D‐values, is symmetrical around 0, indicating that moving from a high‐consumption municipality to a low‐consumption municipality is as common as vice versa. This supports the assumption that moving to a different municipality is exogenous with respect to the consumption of prescribed drugs

## Data Availability

The administrative data used in this study are proprietary and we would be violating confidentiality agreements with government authorities if we distributed the data. The data may be accessed from Swedish national government authorities (Swedish National Board of Health and Welfare, and Statistics Sweden) for researchers that (i) can show that they have ethical approval from an external body for the analyses, and (ii) submit their research plan together with a formal application to the authorities. We are happy to provide detailed information about the process of obtaining the data as well as the full code, going from merging the data sets to analyses.

## Appendix

**TABLE A1:** Results using period 2010–2016—variation across regions

	Model 1	Model 2	Model 3	Model 4	Model 5
Dependent variable	ln(spend+1)	ln(spend+1)	ln(spend+2)	ln(spend+10)	ln(spend)
$\hat{\theta }$ (st.err.)	–0.036 (0.103)	–0.056 (0.103)	–0.060 (0.100)	–0.064 (0.092)	–0.067 (0.100)
95% CI	–0.238; 0.167	–0.259; 0.146	–0.256; 0.136	–0.245; 0.116	–0.263; 0.129
No of ind‐year obs.	263,306	263,306	263,306	263,306	170,871
No of ind.	38,679	38,679	38,679	38,679	35,916
Independent vars.^a^	No	Yes	Yes	Yes	Yes
Years since move	Yes	Yes	Yes	Yes	Yes
Year FE	Yes	Yes	Yes	Yes	Yes

*Note*: Analysis run excluding years pre pharmacy re‐regulation 2009. All fixed effects regressions are run with the sample of migrants over the years 2010–2016. In Model 1, the regression is run without independent variables of individual characteristics. In Model 5, only positive costs are included.

^a^ Independent variables in Model 2–5 include indicators for age‐gender group, individual income, marital status, and the number of children in the household.

**TABLE A2:** Results using period 2010–2016—variation across municipalities

	Model 1	Model 2	Model 3	Model 4	Model 5
Dependent variable	ln(spend+1)	ln(spend+1)	ln(spend+2)	ln(spend+10)	ln(spend)
$\hat{\theta }$ (st.err.)	0.100 (0.036)	0.084 (0.036)	0.081 (0.035)	0.070 (0.033)	0.010 (0.045)
95% CI	0.028; 0.171	0.013; 0.156	0.011; 0.150	0.006; 0.135	–0.078; 0.098
No of ind‐year obs.	508,316	508,316	508,316	508,316	338,677
No of ind.	74,686	74,686	74,686	74,686	69,749
Independent vars.^a^	No	Yes	Yes	Yes	Yes
Years since move	Yes	Yes	Yes	Yes	Yes
Year FE	Yes	Yes	Yes	Yes	Yes

*Note*: Analysis run excluding years pre pharmacy re‐regulation 2009. All fixed effects regressions are run with the sample of migrants across municipalities over the years 2010–2016. In Model 1, the regression is run without independent variables of individual characteristics. In Model 5, only positive costs are included.

^a^ Independent variables in Model 2–5 include indicators for age‐gender group, individual income, marital status, and the number of children in the household.

**TABLE A3:** Additional results from the decomposition analysis

	Model 3	Model 4	Model 5
Dependent variable	ln(spend+2)	ln(spend+10)	ln(spend)
Comparing regions above and below the median			
Difference in average log spending	0.141	0.114	0.044
Place share	0.075	0.087	0.276
95% CI	−0.091; 0.225	−0.077; 0.222	−0.020; 0.582
Individual share	0.925	0.913	0.724
95% CI	0.775; 1.091	0.778; 1.077	0.418; 1.020
Comparing top and bottom quartile of regions			
Difference in average log spending	0.212	0.171	0.069
Place share	0.108	0.113	0.276
95% CI	−0.034; 0.250	−0.025; 0.244	0.007; 0.565
Individual share	0.892	0.887	0.724
95% CI	0.750; 1.034	0.756; 1.025	0.435; 0.993
No of ind‐year obs.	8,242,510	8,242,510	5,863,268
No of ind.	929,711	929,711	881,896

*Note*: The effect shares are estimated in fixed‐effects regressions using the full sample of both migrants and non‐migrants. For each model, the decomposition is estimated comparing regions above/below median spending and regions above p75/below p25 of spending. Confidence intervals are estimated by bootstrapping with 250 repetitions drawn at the individual level and composed by 2.5 and 97.5 percentile of the bootstrap estimates. Each model is run including independent variables (age‐gender group, individual income, marital status, and the number of children in the household) and year fixed effects, region fixed effects, and indicators for the number of years since the move.
