# Supplementary material for: Regional variation in prescription drug spending: Evidence from regional migrants in Sweden
Source: Health Econ. 2022 Jun 16;31(9):1862–77. doi: 10.1002/hec.4552 (PMC9543270; doi:10.1002/hec.4552)
Supplement: Supplementary file 1 — Supporting Information S1 [file HEC-31-1862-s001.docx]

# **SUPPORTING INFORMATION**

### **Regional Variation in Prescription Drug Spending: Evidence from Regional Migrants in Sweden**

## **SKEWNESS IN DRUG SPENDING**

Drug spending per capita is highly right-skewed, with many zeros and a few individuals with very high costs of drugs. The extreme values have a large impact when calculating the regional mean of *log spending*, leading to changes in the ranking of regions compared to the ranking based on raw mean spending.

Table S1 shows the regional mean spending for the regions, listed from lowest to highest. Taking the mean of *log* *spending*, the table shows how the ranking flips around. For example, Kalmar is ranked 5^th^ lowest spending, but in log values, Kalmar is ranked 13^th^, and Gotland is ranked 19^th^ in actual mean spending but 7^th^ lowest in log values. In the event study, we use the difference in mean log spending between regions to approximate the percentage difference. With such a skewed sample, the implication is that we use a negative value of delta for moving from Kalmar to Gotland (5.02–5.08) when in raw values, the mean spending is higher in Gotland compared to Kalmar. In the decomposition analysis, we compare high-consumption to low-consumption regions, so ranking regions is crucial.

To assess the importance of skewness in drug spending, we exclude the top 5% and create a 95 percentile trimmed sample. The bottom panel of Table S1 shows that the issue with ranking differences is reduced in the trimmed sample. Figure S1 shows maps for comparison of the full sample compared to the trimmed sample. Similar to the full sample, the migrants differ from non-migrants (Table S2 and S3). Regional variation is, as expected, smaller in the trimmed sample than the full sample (Figure S2 and Table S4). The results of the event study based on the trimmed sample are similar to the main results of the full sample, estimating a place effect between –0.06 and 0.09 with confidence intervals overlapping zero (Table S5).

**Table S1. Ranking of mean drug spending in full sample vs. trimmed sample**

| **Region** | **Mean drug exp.** | **Ranking of mean drug exp.** | **Ranking of**  **mean log drug exp.** | **Mean log drug exp.** |
| --- | --- | --- | --- | --- |
| **Full sample** |  |  |  |  |
| Västra Götaland | 295.94 € | 1 | 3 | 5.00 |
| Östergötland | 296.27 € | 2 | 1 | 4.88 |
| Jämtland | 296.51 € | 3 | 2 | 4.95 |
| Jönköping | 299.20 € | 4 | 4 | 5.01 |
| Kalmar | 300.17 € | 5 | 13 | 5.08 |
| Örebro | 301.20 € | 6 | 8 | 5.02 |
| Halland | 306.18 € | 7 | 15 | 5.11 |
| Uppsala | 309.46 € | 8 | 5 | 5.01 |
| Blekinge | 312.59 € | 9 | 18 | 5.16 |
| Sörmland | 312.91 € | 10 | 16 | 5.11 |
| Kronoberg | 316.72 € | 11 | 11 | 5.06 |
| Dalarna | 323.54 € | 12 | 12 | 5.08 |
| Gävleborg | 324.79 € | 13 | 17 | 5.16 |
| Stockholm | 326.31 € | 14 | 6 | 5.01 |
| Västerbotten | 329.13 € | 15 | 10 | 5.04 |
| Västmanland | 330.34 € | 16 | 20 | 5.23 |
| Skåne | 332.14 € | 17 | 9 | 5.03 |
| Västernorrland | 333.58 € | 18 | 14 | 5.11 |
| Gotland | 336.63 € | 19 | 7 | 5.02 |
| Norrbotten | 353.35 € | 20 | 21 | 5.25 |
| Värmland | 409.18 € | 21 | 19 | 5.18 |
|  |  |  |  |  |
| **Trimmed sample** | |  |  |  |
| Östergötland | 136.61 € | 1 | 1 | 4.63 |
| Västra Götaland | 138.89 € | 2 | 6 | 4.75 |
| Stockholm | 139.17 € | 3 | 5 | 4.74 |
| Gotland | 139.72 € | 4 | 3 | 4.72 |
| Uppsala | 142.10 € | 5 | 7 | 4.75 |
| Jämtland | 143.56 € | 6 | 2 | 4.68 |
| Kronoberg | 144.16 € | 7 | 11 | 4.78 |
| Jönköping | 144.16 € | 8 | 9 | 4.75 |
| Skåne | 144.17 € | 9 | 4 | 4.74 |
| Halland | 145.06 € | 10 | 16 | 4.85 |
| Örebro | 145.20 € | 11 | 8 | 4.75 |
| Kalmar | 145.48 € | 12 | 13 | 4.82 |
| Blekinge | 145.80 € | 13 | 19 | 4.90 |
| Gävleborg | 148.76 € | 14 | 17 | 4.88 |
| Västerbotten | 149.53 € | 15 | 10 | 4.77 |
| Sörmland | 149.99 € | 16 | 14 | 4.84 |
| Västernorrland | 150.07 € | 17 | 15 | 4.84 |
| Dalarna | 150.39 € | 18 | 12 | 4.80 |
| Västmanland | 153.02 € | 19 | 20 | 4.95 |
| Värmland | 156.36 € | 20 | 18 | 4.90 |
| Norrbotten | 158.70 € | 21 | 21 | 5.00 |

**Figure S1. Maps of mean drug spending**

**Figure S2. Percentage deviation from the national mean in average regional drug spending (trimmed sample)
Note:** Zero on the x-axis represents the national (weighted) mean spending of prescribed drugs per capita per year. The horizontal bars show the percentage deviation in average regional drug spending. The averages are pooled over the years 2007–2016, using the trimmed sample of 910,000 individuals. The trimmed sample's national (weighted) mean was €144 (€1 = 10.5 SEK 2019).

**Table S2. Distribution of drug spending on the individual level**

|  | **Full sample** | **Trimmed sample** | **Of which:**  **Non-migrant** | **Migrants** |
| --- | --- | --- | --- | --- |
| Individuals | 929,711 | 910,639 | 825,391 | 53,248 |
| Ind.-year obs. | 8,242,510 | 7,830,395 | 7,037,543 | 491,378 |
| **Drug spending per capita per year (€)** | | |  |  |
| Mean | 319.25 | 143.62 | 149.59 | 99.85 |
| SD | 2,607.68 | 239.15 | 243.83 | 196.35 |
| Median | 44.29 | 37.24 | 40.19 | 21.90 |
| Min | 0 | 0 | 0 | 0 |
| 25^th^ – 75th perc | 0 - 209.52 | 0 - 162.67 | 0 - 174.48 | 0 - 96.86 |
| Max | 1,617,143 | 1,247 | 1,247 | 1,247 |
| Share obs. with zero costs | 29% | 30% | 30% | 36% |

**Note:** The 95-percentile trimmed sample excludes the top 5% of the sample based on individual-level annual drug spending. The full sample includes 53,620 migrants. A migrant is defined as a person who moves once between regions once in 2008–2015. Individuals who move more than once are excluded from the analysis. Prices 2019 €1 = 10.5 SEK.

**Table S3. Background statistics on non-migrants and migrants in the full and trimmed sample.**

|  | **Full sample** | | **Trimmed sample** | |  |
| --- | --- | --- | --- | --- | --- |
|  | **Non-migrant** | **Migrants** | **Non-migrant** | **Migrants** | |
| Women (%) | 50.77 | 50.66 | 50.61 | 50.53 |  |
| Education level (%) |  |  |  |  |  |
| …Tertiary education | 27.87 | 37.94 | 28.31 | 38.06 |  |
| …Upper secondary school | 43.54 | 38.07 | 43.77 | 38.01 |  |
| …Primary + lower second. school | 27.01 | 22.59 | 26.38 | 22.54 |  |
| …Missing | 1.57 | 1.4 | 1.54 | 1.39 |  |
| Employed (%) |  |  |  |  |  |
| …Yes | 59.46 | 57.49 | 61.41 | 58.00 |  |
| …No | 40.54 | 42.51 | 38.59 | 42.00 |  |
| Marital status (%) |  |  |  |  |  |
| …Married | 44.01 | 24.62 | 43.89 | 24.34 |  |
| …Unmarried | 36.95 | 63.48 | 37.83 | 64.25 |  |
| …Divorced | 11.86 | 9.76 | 11.57 | 9.42 |  |
| …Other | 7.18 | 2.14 | 6.71 | 1.99 |  |
| Children at home (%) |  |  |  |  |  |
| …Yes | 35.21 | 35.6 | 36.52 | 36.04 |  |
| …No | 64.79 | 64.4 | 63.48 | 63.96 |  |
| Age (years) | 49.50  (19.08) | 35.55  (17.08) | 48.72  (18.90) | 35.14  (16.84) | |
| Yearly disp. income ind. (thousand €) | 19.015 (48.669) | 15.711 (24.632) | 19.122 (49.395) | 15.673  (24.832) | |
| Yearly disp. income h.h. (thousand €) | 35.308 (201.438) | 33.124 (40.320) | 35.704 (206.686) | 33.242  (40.608) | |

**Note:** Statistics shown as proportions and for the continuous variables, as mean and standard deviation. The 95-percentile trimmed sample excludes the top 5% of the sample based on individual-level drug spending. A migrant is defined as a person who moves between regions once in 2008–2015.

**Table S4. Distribution of drug spending on the regional level (€)**

|  | Full sample | | Trimmed sample | |
| --- | --- | --- | --- | --- |
| Lowest (of 21 regions) | 295.94 | 136.61 | |  |
| 25^th^ percentile | 301.20 | 143.56 | |  |
| Median | 316.72 | 145.20 | |  |
| 75^th^ percentile | 330.34 | 149.99 | |  |
| Highest | 409.18 | 158.70 | |  |
|  |  |  | |  |
| Mean (unweighted) | 321.25 | 146.23 | |  |
| Standard deviation | 25.69 | 5.69 | |  |
|  |  |  | |  |
| Ratio highest/lowest | 1.38 | 1.16 | |  |
| Ratio 75^th^/25^th^ | 1.10 | 1.04 | |  |
| Coefficient of variation | 0.08 | 0.04 | |  |

**Note:** Regional mean spending of prescribed drugs per capita per year. The averages are pooled over the years 2007–2016. The coefficient of variation is calculated as the ratio of the standard deviation to the mean. Prices 2019 €1 = 10.5 SEK.

**Table S5. Results from event study regressions (trimmed sample)**

|  | Model 1 | Model 2 | Model 3 | Model 4 | Model 5 |
| --- | --- | --- | --- | --- | --- |
| Dependent variable | ln(spend+1) | ln(spend+1) | ln(spend+2) | ln(spend+10) | ln(spend) |
|  |  |  |  |  |  |
| $\hat{\boldsymbol{\theta}}$ (st.err.) | 0.089 **(**0.088) | 0.094 **(**0.088) | 0.082 **(**0.085) | 0.050 **(**0.080) | -0.056 **(**0.081) |
| 95% C.I. | -0.083; 0.261 | -0.077; 0.266 | -0.086; 0.249 | -0.106; 0.206 | -0.215; 0.103 |
| No of ind-year obs. | 491,378 | 491,378 | 491,378 | 491,378 | 313,331 |
| No of ind. | 53,248 | 53,248 | 53,248 | 53,248 | 50,899 |
| Independent vars. | No | Yes | Yes | Yes | Yes |
| Years since move | Yes | Yes | Yes | Yes | Yes |
| Year FE | Yes | Yes | Yes | Yes | Yes |

**Note:** All fixed effects regressions are run with the 95-trimmed sample of 53,248 migrants over the years 2007-2016. In Model 1, the regression is run without independent variables of individual characteristics. In Model 5, zero cost observations are excluded. Independent variables in Model 2-5 include indicators for age-gender group, individual income, marital status, and the number of children in the household.
